# Expression and cellular localisation of *Trypanosoma cruzi* calpains

**DOI:** 10.1590/0074-02760200142

**Published:** 2020-10-12

**Authors:** Vítor Ennes-Vidal, André Nóbrega Pitaluga, Constança Felícia De Paoli de Carvalho Britto, Marta Helena Branquinha, André Luis Souza dos Santos, Rubem Figueiredo Sadok Menna-Barreto, Claudia Masini d’Avila-Levy

**Affiliations:** 1 Fundação Oswaldo Cruz-Fiocruz, Instituto Oswaldo Cruz, Laboratório de Estudos Integrados em Protozoologia, Rio de Janeiro, RJ, Brasil; 2 Fundação Oswaldo Cruz-Fiocruz, Instituto Oswaldo Cruz, Laboratório de Biologia Molecular de Parasitas e Vetores, Rio de Janeiro, RJ, Brasil; 3 Fundação Oswaldo Cruz-Fiocruz, Instituto Oswaldo Cruz, Laboratório de Biologia Molecular e Doenças Endêmicas, Rio de Janeiro, RJ, Brasil; 4 Universidade Federal do Rio de Janeiro, Instituto de Microbiologia Paulo de Góes, Laboratório de Estudos Avançados de Microrganismos Emergentes e Resistentes, Rio de Janeiro, RJ, Brasil; 5 Universidade Federal do Rio de Janeiro, Instituto de Química, Programa de Pós-Graduação em Bioquímica, Rio de Janeiro, RJ, Brasil; 6 Fundação Oswaldo Cruz-Fiocruz, Instituto Oswaldo Cruz, Laboratório de Biologia Celular, Rio de Janeiro, RJ, Brasil

**Keywords:** cysteine peptidase, ultrastructure, Chagas disease, trypanosomatid

## Abstract

**BACKGROUND:**

Calpains are present in almost all organisms and comprise a family of calcium-dependent cysteine peptidases implicated in crucial cellular functions. *Trypanosoma cruzi*, the causative agent of Chagas disease, presents an expansion on this gene family with unexplored biological properties.

**OBJECTIVES:**

Here, we searched for calpains in the *T. cruzi* genome, evaluated the mRNA levels, calpain activity and the protein expression and determined the cellular localisation in all three parasite life cycle forms.

**METHODS/FINDINGS:**

Sixty-three calpain sequences were identified in *T. cruzi* CL Brener genome, with fourteen domain arrangements. The comparison of calpain mRNA abundance by quantitative polymerase chain reaction (qPCR) revealed seven up-regulated sequences in amastigotes and/or bloodstream trypomastigotes and five in epimastigotes. Western Blotting analysis revealed seven different molecules in the three parasite forms, and one amastigote-specific, while no proteolytic activity could be detected. Flow cytometry assays revealed a higher amount of intracellular calpains in amastigotes and/or trypomastigotes in comparison to epimastigotes. Finally, ultrastructural analysis revealed the presence of calpains in the cytoplasm, vesicular and plasma membranes of the three parasite forms, and in the paraflagellar rod in trypomastigotes.

**CONCLUSION:**

Calpains are differentially expressed and localised in the *T. cruzi* life cycle forms. This study adds data on the calpain occurrence and expression pattern in *T. cruzi*.

Calpains (EC 3.4.22.17, Clan CA, family C02) comprise a family of cytosolic calcium-dependent cysteine peptidases defined by a CysPc motif, a well-conserved cysteine peptidase domain. These proteins have a limited activity and act to modulate or transform the substrate, and are therefore known as “modulator peptidases”. Calpains were discovered in 1964 in mammals and homologs were later described in insects, nematodes, plants, fungi, protozoan parasites such as trypanosomatids, and bacteria. There are 15 calpains in the human genome, and a genetic mutation in each of these genes causes lethality or disorders known as calpainopathies. Calpain inhibitors were already used in clinical trials to treat Duchenne muscular dystrophy and Alzheimer’s disease.^1^ ^,^ ^2^

The Trypanosomatidae family (class Kinetoplastea) encompasses parasitic protists, some of which are responsible for important humans diseases including Chagas disease caused by *Trypanosoma cruzi*.^3^ The protozoan undergoes profound morphological changes during its development in a complex life cycle involving mammalian and invertebrate hosts. There are major morphological stages: an obligate proliferative mammalian intracellular form, namely amastigote; the infective trypomastigote present in the both hosts; and the proliferative insect form, namely epimastigotes.^4^

The clinical course of Chagas disease usually comprises an acute and a chronic phase. The former is asymptomatic in most cases, but patients can develop chronic disease, which in up to 30-40% of cases is characterised by cardiomyopathy, arrhythmias, megaviscera, and more rarely, polyneuropathy and stroke.^4^ Moreover, orally acquired acute Chagas disease can be potentially fatal. The available chemotherapy was empirically discovered and presents common side effects and limited efficacy. Therefore, the search for more effective drugs is still an urgency.^5^ Considering that the challenge for the introduction of new compounds for neglected diseases is more economical than biological,^6^ a repurposed approach with compounds approved for human usage could be an alternative shortcut for a chemotherapeutic treatment for trypanosomatids’ infections.^7^ Over the past few years, our research group has been reporting the effects of the calpain inhibitor MDL28170 (inhibitor III, Z-Val-Phe-CHO) against all *T. cruzi* evolutive stages.^8^

Mammals and most organisms have few calpain genes in their genomes. However, in trypanosomatids this reality is quite different. In 2005, Ersfeld and colleagues^9^ described the presence of a large and diverse family of 24 calpain sequences in *T. cruzi*, in addition to 18 sequences in *T. brucei* and 27 in *Leishmania major*. Genome sequencing and assemblage improvements are paving the road for protein families’ analysis. For instance, H49, a classic virulence factor of *T. cruzi*, was shown to be a member of the calpain family.^10^ Trypanosomatids’ calpains started to be unveiled in the last 15 years, and there is yet a lot to be explored. Here, we screened *T. cruzi* genome to identify and classify calpain sequences and their domain composition, and compared the gene expression among epimastigotes, amastigotes and trypomastigotes. Calpain activity was screened by zymography and fluorometry. The protein expression pattern and the ultrastructural localisation in each life cycle stage were assayed by Western Blotting and transmission electron microscopy, respectively. These data can help to determine the functions of this highly diverse multigene family in this parasite.

## MATERIALS AND METHODS

*Calpain search in* T. cruzi *genome, conserved domain analysis, sequence selection and primers design* - Protein sequences of *T. cruzi* CL Brener Esmeraldo and Non-Esmeraldo strains annotated as calpains were retrieved from Tritryp Database (https://tritrypdb.org). These proteins were locally analysed by Simple Modular Architecture Research Tool (SMART) for the presence of calpain domains DUF1935, CysPc and CBSW in InterPro and Pfam databases. In addition, an HMM model was created with a wide range of annotated calpains and an HMMsearch was performed in *T. cruzi* genome (GenBank ID 25). Sequences containing less than 100 amino acid residues and domains with e-value higher than 10^-3^ were removed from the analysis. Gene-specific primers of sequences harboring the calpain proteolytic core (CysPc) were designed using Primer3Plus to amplify a 90 to 120 bp fragment for quantitative polymerase chain reaction (qPCR) analysis [Supplementary data (Table I)]. The predicted molecular masses of calpain sequences were calculated in Bioinformatics.org (http://www.bioinformatics.org/sms/prot_mw.htm).

*Parasite cultivation and amastigote and trypomastigote isolation* - *T. cruzi* epimastigotes from Y strain (COLPROT 106) were obtained from Coleção de Protozoários da Fundação Oswaldo Cruz (FIOCRUZ-COLPROT, http://colprot.fiocruz.br). To reach mid-log phase, epimastigotes were routinely maintained at 28ºC for five days in liver infusion tryptose medium (LIT; 5.0 g/L liver infusion, 5.0 g/L tryptose, 4.0 g/L NaCl, 0.4 g/L KCl, 4.3 g/L Na_2_HPO_4_.H_2_O, 2.0 g/L glucose D+) supplemented with 10% heat-inactivated fetal bovine serum (Sigma-Aldrich, St. Louis, MO, United States) and 0.1% hemin. Separation of trypomastigotes and amastigotes was carried out using Vero cells, as detailed elsewhere.^11^ Briefly, Vero cells were infected with mice-derived bloodstream trypomastigotes in a 10:1 parasite/host cell ratio. Infected cells were maintained at 37ºC in 5% CO_2_ atmosphere. After five days, the supernatant was collected, centrifuged at 500 × g for 5 min, and incubated at 37ºC for 30 min for the migration of trypomastigotes into the supernatant. The amastigotes remained in the pellet. Experiments were carried out in accordance with protocols approved by the Institutional Animal Care and Use Committee at Instituto Oswaldo Cruz of Fiocruz (CEUA LW 16/13).

*Gene expression comparison between* T. cruzi *life cycle forms* - Total RNA from epimastigote, amastigote and trypomastigote forms was extracted using TRIzol^®^ reagent (Invitrogen, Carlsbad, CA, United States) according to the manufacturer’s instructions. RNA samples were treated with DNAse I (Sigma-Aldrich) to remove any contaminating DNA, analysed for purity and quantified in a spectrophotometer. The cDNA synthesis was performed with SuperScriptIII kit (Applied Biosystems, Foster City, CA, United States) using oligo-dT primers. The specificity of each designed primer was confirmed by sequencing the amplified products in the Sanger ABI 3730 Sequencing. The PCR products sequences were evaluated against NCBI *nr* database using BLASTn. For qPCR, cDNA (~800 ng/μL) was diluted 10 times and used in 20 µL Go-Taq PCR Master Mix (Promega, Madison, WI, United States) reaction and primers in the ABI Prism 7500 FAST (Applied Biosystem). The relative gene expression was determined using comparative CT values. ΔCT of the target sequence was obtained as the difference on the CT value from endogenous control glycosomal glyceraldehyde-3-phosphate dehydrogenase (*gGAPDH*) gene. The constitutive *18S* gene was analysed in parallel to improve data confidence and provided similar results (data not shown). The ΔΔCT value of each gene was calculated pair-to-pair between the three life cycle forms - after validation of primers efficiencies of at least 95% -, and epimastigotes were regarded as the reference. The relative expression was then reported as 2^−ΔΔCT^.^12^

*Calpain enzymatic activity screening* - Whole parasite cellular extracts from *T. cruzi* epimastigotes were obtained by ressuspending the cells in lysis buffer (80 mM HEPES pH 7.5, 150 mM NaCl_2_, 1 mM MgCl_2_, 1% NP-40, 3 mM EGTA, 1 U/μL DNase) during 5 min in an ice-bath. The crude extract was centrifuged at 4ºC and the soluble and insoluble fractions were separated by centrifugation (14,000 × *g*). Calpain activity of both fractions were assayed simultaneously by gel zymography and fluorimetry. Approximately 40 µg of each fraction were submitted to 10% polyacrylamide gel electrophoresis (PAGE) incorporated with 0.2% gelatin in an ice-bath under non-reducing conditions. After running, the gels were washed four times with 25 mM Tris-HCl, pH 7.5 (reaction buffer), and incubated with 0.25, 1.0 or 2.0 mM CaCl_2_ at 37ºC for 72 h, stained in Coomassie Blue R-250 in methanol-acetic acid-water and destained in the same solvent. Alternatively, one gel was incubated in the reaction buffer with different concentrations of CaCl_2_ (from 0.062 to 2.0 mM) in the presence of 25 μM N-Suc-Leu-Leu-Val-Tyr-7-amido-4-Methilcoumarin, a substrate of the CA clan of cysteine peptidases (www.merops.sanger.ac.uk). After 30 min incubation, free 7-Amino-4-methylcoumarin (AMC) was observed in a ultraviolet transilluminator, as described.^13^ Whole cellular kidney extracts from mice were used as a positive control.

*Flow cytometry analysis* - Parasites from the three distinct parasite life cycle forms (1.0 × 10^6^ cells) were processed and analysed for flow cytometry as previously described.^8^ Briefly, 0.4% paraformaldehyde-fixed parasites, permeabilised or not with 0.01% Triton X-100, were incubated at room temperature for 2 h with anti-tritryp-calpain polyclonal antibody (1:250 dilution).^14^ Cells were washed three times to remove non-adhered antibodies and incubated with Alexa 488 goat anti-rabbit IgG (1:750 dilution) for 1 h at room temperature. Data acquisition and analysis were performed on a flow cytometer equipped with a 15 mW argon laser emitting at 488 nm (FACSCalibur, BD Bioscience, USA). The omission of the primary antibody was used as a negative control. Each experimental population was then mapped by using a two-parameter histogram of forward-angle light scatter versus side scatter. The mapped population (*n* = 10,000) was then analysed for log green fluorescence by using a single parameter histogram, and the mean fluorescence intensity (MFI) of each experimental system was divided by MFI from autofluorescence controls to obtain the variation index.

*Identification of calpains by Western Blotting* - *T. cruzi* life cycle forms (1.0 × 10^8^ cells) were collected by centrifugation at 3,000 × *g* for 10 min at 4ºC and washed three times with cold phosphate buffered saline (PBS, pH 7.2). The cells were lysed with 100 µL of sodium dodecyl sulfate-PAGE (SDS-PAGE) sample buffer (62 mM Tris-HCl pH 6.8, 2% SDS, 25% glycerol, 0.01% bromophenol blue and 1 mM β-mercaptoethanol) supplemented with cOmplete^™^ Mini Protease Inhibitor Cocktail (Roche, Basel, Switzerland). Proteins were separated in 10% SDS-PAGE under reducing conditions and the polypeptides were electrophoretically transferred (100 V/300 mA) at 4ºC for 2 h to nitrocellulose membranes and blocked in 10% low-fat dried milk dissolved in PBS containing 2% Tween 20 (TBS/Tween) overnight at 4ºC, and then washed with the blocking solution and incubated with anti-tritryp-calpain antibody (1:500 dilution) for 2 h. The secondary peroxidase-conjugated goat anti-rabbit immunoglobulin G (1:1500 dilution) was used followed by chemiluminescence immunodetection. An anti-β-actin polyclonal antibody produced in rabbit (Rhea Biotech, Brazil) (1:5,000 dilution) was used as a loading control. The relative molecular masses of the reactive polypeptides were calculated by comparison with the mobility of SDS-PAGE standards and the densitometric analysis was performed using ImageJ software.

*Immunocytochemistry by transmission electron microscopy* - Briefly, *T. cruzi* life cycle forms (1.0 × 10^6^ cells/mL) were washed three times in PBS and fixed with 2% paraformaldehyde, 0.1% glutaraldehyde and 0.02% picric acid in 0.1 M Na-cacodylate buffer (pH 7.2) for 40 min. Parasites were then dehydrated in an ascending methanol series (30-100%) and embedded in MonoStep™ Lowicryl K4M Polar Embedding Media (Polysciences, Inc., USA). The inclusions polimerisation was performed by indirect UV-irradiation at -20ºC for seven days. The ultrathin sections obtained were incubated with a 1:20 dilution of polyclonal rabbit anti-tritryp-calpain antibody for 2 h, followed by labeling with a 1:40 dilution of the secondary anti-rabbit-gold (10 nm) antibody (Sigma-Aldrich) for 1 h. After the immunolabeling, ultrathin sections were stained with uranyl acetate and examined in Jeol JEM1011 transmission electron microscope at Plataforma de Microscopia Eletrônica, IOC, FIOCRUZ.

*Statistical analysis* - All experiments were repeated at least three times and performed in triplicate and the results shown represent the mean and standard deviation of the mean. When appropriate, representative images of the experiments are shown. The data were analysed statistically by Student’s *t* test using GraphPad Prism Version 5.00 software. *P* values of 0.05 or less were considered statistically significant.

## RESULTS AND DISCUSSION

*A massive expansion of calpain sequences in* T. cruzi *genome* - Since its first report by Guroff in 1964,^15^ a wide range of calpain homologs has been described in almost all eukaryotic organisms and some bacteria.^2^ The proteins encoded by these sequences have the most varied structures and domain combinations, ranging from classical domains of the calpain family, such as the peptidase core domain (CysPc) and the calpain-type beta-sandwich domain (CBSW, formerly called “C2-domain-like-C2L”), to several other domains with the most varied functions and origins. Here, we found 63 calpain sequences in *T. cruzi* genome from CL Brener Esmeraldo and Non-Esmeraldo distributed in 11 different chromosomes [Supplementary data (Table II)]. The domain arrangements of these sequences comprise both the conserved CysPc and CBSW domains. Others domains were also identified, like Domain of Unknown Function 1935 (DUF1935), regular structure consisting of similar repeats (RNI-like) and repeat rich regions (RPT), the latter already reported by Ersfeld and colleagues (Table I). These data are in agreement with our previous work with *Leishmania braziliensis*, whereas the presence of a large diversity of domain arrangement was revealed in 34 calpain-related sequences distributed in 13 different chromosomes.^14^ However, microtubule interacting and trafficking domain (MIT) was not found associated with *T. cruzi* calpains.

**TABLE I:** Calpain sequences distribution in *Trypanosoma cruzi* genome

Domain architectures	Number of sequences	Chromosomes	Predicted molecular masses	Sequences with conserved catalytic triad (C,H,N)
DUF1935	25	9, 11, 31, 40	12.81 - 40.31	-
fragmented CysPc	1	31	24.7	-
CysPc	4	31, 35, 39, 40	19.13 - 85.46	-
DUF1935, CysPc	16	8, 9,17, 31	78.11 - 161.78	2 (TcCLB.503855.40 and TcCLB.510337.20)
CysPc, CBSW	4	14, 39	65.54 - 135.52	2 (TcCLB.506227.130 and TcCLB.511847.10)
RNI-like, CysPc, CBSW	3	32, 40	88.15 - 116.16	-
RNI-like, CBSW, CysPc, RPT1	2	5	179.71	-
RPT1, CysPc, CBSW	1	39	154.68	-
CysPc, CBSW, RPT1	1	39	167.65	-
CysPc, RPT3, RPT2	1	39	200.83	-
2×RPT1, CysPc	1	39	145.25	-
2×RPT1, CysPc, CBSW	1	39	132.88	-
KISC, 3×ARM, CBSW	1	17	150.32	-
CysPc, CBSW, CysPc, RPT2, CysPc, CBSW	1	39	519.41	-

Calpain sequences were retrieved from *T. cruzi* genome, and their chromosome location and conservation of the catalytic triad are indicated. The domain combinations were obtained from Simple Modular Architecture Research Tool (SMART). The predicted molecular mass was calculated in http://www.bioinformatics.org/sms/prot_mw.html. ARM: armadillo/beta-catenin-like repeats; CBSW: calpain-type alpha-beta-sandwich domain; CysPc: proteolytic core domain; KISC: kinesin domain; DUF1935: conserved N-terminal calpain-related domain from trypanosomatids; RPT: repeated domain found in de-ubiquitinating proteins; RNI-like: regular structure consisting of similar repeats, 2× and 3× indicate the number of times that a domain appears in the sequence. Fragmented CysPc stands for short amino acid sequences from the catalytic domain.

We identified four domains in either Bacteria or Archea, and two exclusively found in eukaryotes by searching protein family databases (Table II). The novelty of calpain arrangements found in trypanosomatids could have taken place by the addition of both ancient and novel domains to the N- or C- terminus of CysPc with variable types, numbers and orders. Accordingly, to investigate whether the eukaryotic CysPc paralogs derived from bacteria once or many times in unicellular eukaryotes, Zhao and co-workers^2^ analysed several prokaryote CysPc domains by phylogenetic reconstruction. The resulting tree revealed that bacterial paralogs are weakly recovered as a monophyletic group with affinity to one eukaryotic CysPc clades, indicating that all eukaryote CysPc variants may have evolved from a single bacterial paralog. The distribution of the domains found here and in the Eukarya, Bacteria and Archea, as well as their associated functions, are summarised in Table II.

**TABLE II:** Identified domains from *Trypanosoma cruzi* calpains and their distribution in the three domains of life

Abbreviation	Description	Eukarya	Bacteria	Archaea	Function	Reference
ARM	Armadillo/beta-catenin-like repeats	*	*	*	Mediates interaction of beta-catenin with intracellular signals, and regulates cytoskeletal process	^29^
CBSW	Calpain-type beta-sandwich domain	*			Substrate recognition and regulation of calpain activity	Reviewed in^(1)^
CysPc	Calpain catalytic core domain	*	*		Signal transduction, cell division, apoptosis and differentiation	Reviewed in^1^
DUF1935	Domain of unknown function (SMP-1)	*			Target flagellar localisation	^16^
KISC	Kinesin motor domain	*			Intracellular traffic by microtubules, and cellular division	Reviewed in^14^
RPT^1^	Repeat rich regions	*	*	*	Confers rigidity to protein structure	Reviewed in^14^
RNI-like	Regular structure consisting of similar repeats	*	*		Cellular adhesion	Reviewed in^14^

*: indicates calpain domain presence; ^1^: repeat rich regions do not correspond to traditionally domains, but they are important to conservate the protein structure.^30^

Among the 63 calpain sequences found in *T. cruzi* genome, only 35 presented the classical calpain domain CysPc in combination or not with other domains. None of them corresponds to a classical calpain since the penta-EF-hand (PEF) domain was not detected. Moreover, the absence of amino acid residues essential for the catalytic activity (C,H,N) and the overall degree of 20-30% identity between the sequences suggest that the majority of *T. cruzi* calpains does not have proteolytic activity. Only four calpains have the conserved catalytic triad: TcCLB.503855.40, TcCLB.510337.20, TcCLB.506227.130 and TcCLB.511847.10 (Table I). One small sequence (TcCLB.511333.10) in the chromosome 31 presents a fragmented CysPc domain of just 26 amino acids and, although annotated as a calpain-like protein, it was not considered to further analysis due to its small size and low identity (e-value > 10^-3^).

The *T. cruzi* genome has 41 predicted sequences with DUF1935, standing as the unique domain in 25 of these sequences distributed across chromosomes 9, 11, 31 and 40. This small domain is exclusively found in trypanosomatids and comprises some protein sequences with an average length of 200 amino acids approximately. DUF1935 was reported by Ersfeld and colleagues (2005)^9^ as an unique domain of small sequences exclusively found in trypanosomatids, and called as small kinetoplastid calpain-related proteins (SKCRPs). At the same year, another study identified one of these molecules as a diacylated membrane protein from *L. major*, named as small myristoylated protein-1 (SMP-1), and reported the role of SMP-1 as a signal to a specific flagellar localisation.^16^ Here, small sequences were not considered for further investigations, although two proteomic studies reported the protein expression of SKCRPs in epimastigotes, metacyclic and bloodstream trypomastigotes.^17^ ^,^ ^18^

As previously observed in a calpain screening in *L. braziliensis*,^14^ *T. cruzi* also presents in chromosome 17 an ortholog sequence that possesses a kinesin motor domain associated to three armadillo/beta-catenin-like repeats (ARM) positioned before the CBSW domain (TcCLB.511307.10). In *T. brucei*, this orphan kinesin (KIN-E) enrichment at the flagellar tip depends on the CBSW domain, and the KIN-E depletion in the trypomastigote form causes major morphology changes generating epimastigote-like parasites.^19^ Therefore, the kinesin calpain was included in the gene expression analysis.

Several RPT domains (RPT1, RPT2 and RPT3) were associated with CysPc calpains. These sequences are related to repeat-rich H49 proteins, which were previously identified as members of the calpain family.^10^ Eight sequences carrying the 204-bp repeats of H49 proteins associated with CysPc were found and referred as H49/calpains, but not all of them have a calpain-associated domain. Here, six sequences annotated as “calpain” with RPT regions were found in chromosome 39 of CL Brener genome associated with a CysPc domain, and only two sequences in chromosome 5 [Supplementary data (Table II)]. Due to the well-defined roles played by RPT-associated calpains, these molecules were not the focus of our subsequent investigations by qPCR.

*Differential calpain gene expression profile among* T. cruzi *epimastigotes, amastigotes and trypomastigotes* - Trypanosomatids, such as *T. cruzi*, have gene expression regulation peculiarities. The mRNAs transcribed in constitutive polycistronic units are not necessarily related to specific functions, and mature mRNA levels may vary between transcripts. Thus, trypanosomatids’ gene expression may be regulated post-transcriptionally, with an important role of the 3’-UTR and 5’-UTR sequences in the life decay and processing of these molecules.^20^ In this sense, it has been shown a link between differential expression levels of calpains’ transcripts and protein expression during trypanosomatids differentiation.^14^ ^,^ ^21^ ^,^ ^22^ ^,^ ^23^

Here, we evaluated by qPCR the relative gene expression of the calpains with the most conserved domain architecture, such as the presence of the CysPc, and removed from the analysis the repeat-rich and small sequences. From the 26 selected sequences, 20 were identical, which precluded the design of specific primers, resulting in the exclusion of 10 sequences from the subsequent analysis. In order to perform the gene expression analysis, 16 calpain-specific primers and two reference genes (*gGAPDH* and *18S*) were designed [Supplementary data (Table I)].

For the biological interpretation of the calpain sequences expression levels, we ordered the sequences in seven different groups due to their modulation pattern (Table III). Our analysis revealed two calpain sequences up-modulated in the amastigote and trypomastigote in relation to the epimastigote form (■), one highly expressed only in the amastigotes (○), three significantly up-modulated in amastigotes but down regulated in trypomastigote (▲), and one up-modulated only in trypomastigotes ( ◊ ). In addition, five sequences were significantly up-modulated in the insect form in comparison to the clinical relevant forms (●), and two calpains showed not significant modulation between epimastigotes and amastigotes, reporting a reduced expression in trypomastigotes (□). Only two sequences showed a constitutive expression between the three different evolutionary forms of *T. cruzi* (♦).

**TABLE III:** Differential gene expression levels of calpain sequences from *Trypanosoma cruzi* epimastigote, amastigote and trypomastigote forms

Group	Sequence ID	Domain architecture	Gene expression ratio		
			Epimastigotes	Amastigotes	Trypomastigotes
■	TcCLB.511307.10	KISC, 3×ARM, CBSW	1 ± 0,15	2.6 ± 0,01	13.8 ± 0,06
	TcCLB.509237.140	DUF1935, CysPc	1 ± 0,04	5.1 ± 0,93	5.5 ± 0,18
○	TcCLB.504107.10	CysPc, CBSW	1 ± 0,12	56.9 ± 1,44	0.5 ± 0,13
▲	TcCLB.511269.70	RNI-like, CysPc, CBSW	1± 0,07	8.9 ± 0,60	0.3 ± 0,10
	TcCLB.511507.70	RNI-like, CysPc, CBSW	1 ± 0,07	2.5 ± 0,30	0.3 ± 0,60
	TcCLB.511333.4	DUF1935, CysPc	1 ± 0,02	1.9 ± 0,18	0.2 ± 0,03
◊	TcCLB.508999.200	DUF1935, CysPc	1 ± 0,03	0.8 ± 0,20	4.3 ± 0,23
●	TcCLB.509237.120	DUF1935, CysPc	1 ± 0,08	0.3 ± 0,05	0.4 ± 0,01
	TcCLB.510337.20	DUF1935, CysPc	1 ± 0,04	0.1 ± 0,02	0.1 ± 0,01
	TcCLB.508999.230	DUF1935, CysPc	1 ± 0,04	0.2 ± 0,03	0.4 ± 0,07
	TcCLB.511847.10	CysPc, CBSW	1 ± 0,03	0.2 ± 0,01	0.2 ± 0,03
	TcCLB.506563.200	DUF1935, CysPc	1 ± 0,07	0.2 ± 0,01	0.4 ± 0,06
□	TcCLB.508999.190	DUF1935, CysPc	1 ± 0,08	0.7 ± 0,10	0.4 ± 0,11
	TcCLB.511329.10	CysPc	1 ± 0,03	1.0 ± 0,11	0.2 ± 0,11
♦	TcCLB.511727.30	CysPc	1 ± 0,05	1.3 ± 0,01	0.8 ± 0,06
	TcCLB.509013.19	CysPc, CBSW	1 ± 0,07	1.0 ± 0,03	0.9 ± 0,23

Transcripts were validated by quantitative polymerase chain reaction (qPCR) and the resulting ratios between the three life cycle forms are given. Epimastigotes were fixed as the reference sample. Bold numbers indicate calpain sequences that differed statistically significantly in relation to epimastigotes (p < 0.05). Sequences were distributed in groups according to their regulation pattern: ■ up-modulated in the amastigotes and trypomastigotes; ○ up-modulated in amastigotes; ▲ up-modulated in amastigotes and down-modulated in trypomastigotes; ◊ up-modulated in trypomastigotes; ● up-modulated in epimastigotes; down-modulated in trypomastigotes; and ♦ constitutive expression. TritrypDB accession numbers and the domain architecture of each calpain sequence are given. Glycosomal glyceraldehyde-3-phosphate dehydrogenase (*gGAPDH*) and 18S were used as endogenous controls. The mean of at least four independent experiments, performed in triplicate, and the standard error of the mean are shown.

Interestingly, our analysis revealed a 56.9 times higher expression of one calpain, *TcCLB.504107.10*, in amastigotes compared to epimastigotes, a relative value that increased two times when compared to trypomastigote forms. Notwithstanding, a differential expression profile of calpains has been reported in distinct parasite life cycle forms or in parasites submitted to either nutritional or drug stressors.^14^ ^,^ ^21^ ^,^ ^22^ ^,^ ^23^ ^,^ ^24^ In *Leishmania donovani*, a comparative proteomic screening between antimonial-resistant and -sensitive strains revealed that a calpain-like molecule (containing a DUF1935 domain), is down-regulated in the resistant strain, and could modulate the susceptibility to antimonials and miltefosine by interfering with drug-induced programmed cell death (PCD) pathways.^24^ It was demonstrated by qPCR an up-modulation of a *T. cruzi* calpain expression (*TcCLB.508999.200*), which was 2.5 times higher in epimastigotes submitted to nutritional stress preceding metacyclogenesis in relation to parasites in nutrient-rich medium.^23^ Here, *TcCLB.508999.200* showed a 4.3 times higher expression in bloodstream trypomastigotes than in epimastigotes grown in nutrient-rich axenic medium.

In *T. brucei*, some calpains have well established functions and differences in calpain gene expression were already reported between procyclic and bloodstream trypomastigote forms. A paralog variant of CAP5.5, named *CAP5.5V*, has a 15-fold increased expression in metacyclic trypomastigotes.^21^ *CAP5.5* and *CAP5.5V* are related to the correct morphogenesis of *T. brucei*, as observed in ultrastructural studies of RNA*i*-induced parasites for these two proteins. At least two calpain transcripts from bloodstream trypomastigotes and three others from the procyclic forms of the parasite have a significant modulation of expression.^22^ The specific regulation of transcripts suggests that calpains plays critical roles in trypanosomatids. This differential expression could be associated with the differentiation process and the development of each life cycle and its specific functions, such as, the locomotion and adhesion to the digestive tract and/or escape of the invertebrate host immune system in epimastigotes and infection and multiplication in the vertebrate host in trypomastigotes and amastigotes.

Calpain biological functions have always been associated with enzymatic activity.^1^ However, in trypanosomatids this direct correlation has never been demonstrated. Under the conditions assayed here, no calpain activity was detected in epimastigotes (data not shown). Accordingly, no calpain activity was demonstrated in *T. cruzi* H49 calpain family members.^10^ Interestingly, an endosymbiont-bearing insect trypanosomatid, *Angomonas deanei*, produces a proteolytically active calpain.^25^ The sequences that possess the conserved catalytic triad in *T. cruzi* are transcribed and showed altered levels of transcripts among the life cycle forms of *T. cruzi* with an up-modulation in epimastigotes and constant levels between trypomastigote and amastigotes (Table I). The understanding of the molecular mechanisms of calpain gene expression regulation, as well as the role played by the proteolytic activity of calpains in these organisms, is critical to validate these molecules as potential novel targets for searching a selective and more efficient treatment against the diseases caused by these protists.^7^

**Fig. 1:**
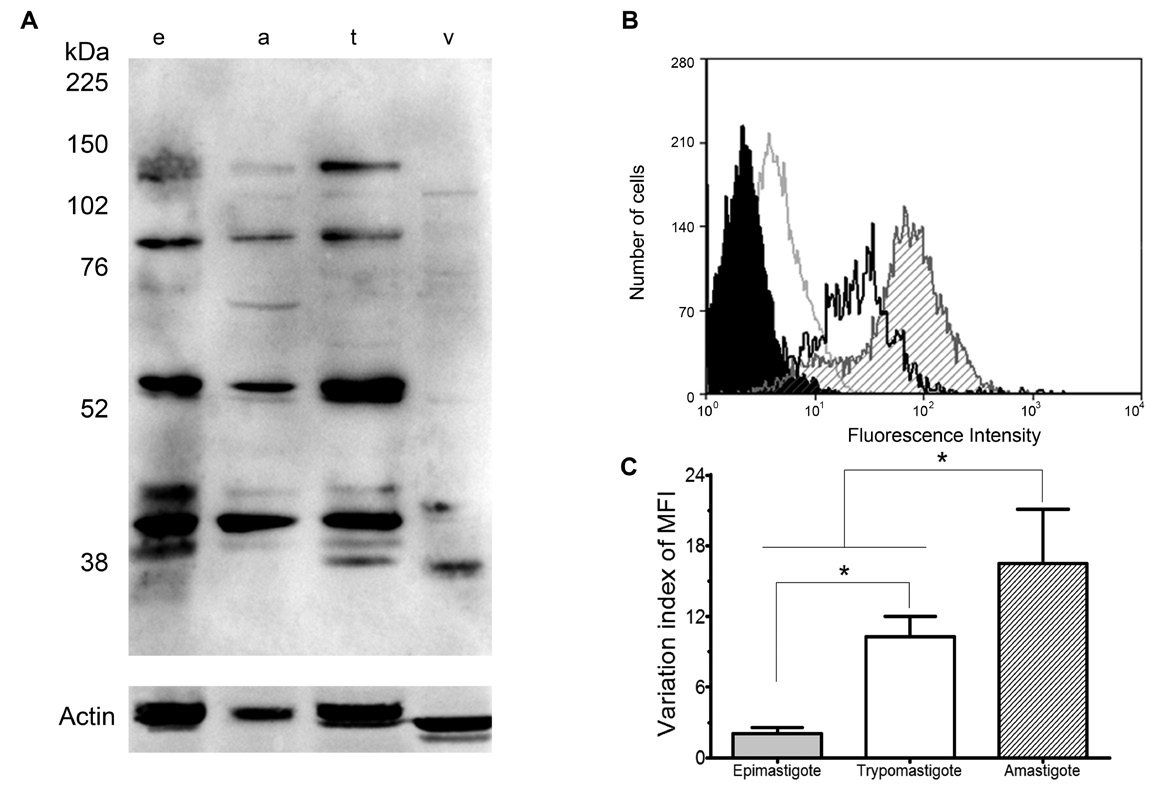
protein expression pattern of calpains recognised by the anti-tritryp-calpain antibody in the three *Trypanosoma cruzi* life cycle forms. (A) Immunoblotting of total cellular extracts of epimastigotes (e), amastigotes (a) and trypomastigotes (t) of *T. cruzi*, and extracts of Vero cells (v). The membrane was incubated with anti-tritryp-calpain at 1:250. The relative molecular mass of sodium dodecyl sulfate-polyacrylamide gel electrophoresis (SDS-PAGE) protein standards is shown in the left. An anti-actin antibody was used as a control for sample loading in the blots. (B) Flow cytometry analysis of epimastigotes (grey line), amastigotes (dashed fill) and trypomastigotes (black line) labeled with anti-tritryp-calpain antibody. Autofluorescence controls using only with the secondary-Alexa 488 antibody generated similar curves to that observed in the autofluorescence control (data not shown), and just one control is represented (black fill). Representative data of the 10,000 cells analysis from one out of three experiments are shown. (C) The variation index of the mean fluorescent intensity (MFI) from each life cycle form was obtained by the division of the MFI from labeled parasites by the autofluorescence controls from the flow cytometric analysis. The bars indicate the standard deviation of the mean, and * indicates statistically significant difference between each life cycle MFI (p < 0.05).

*Protein expression pattern of calpains in the three distinct* T. cruzi *forms* - Recently, we selected a consensus polypeptide (LEKAYAKLHGSY) in order to produce a polyclonal antibody capable of recognising proteins comprising the conserved CysPc domain to be used as a tool for assessing the global profile in CysPc-containing calpains.^14^ Here, Western Blotting analysis of *T. cruzi* extracts with the anti-calpain antibody revealed a complex pattern of proteins recognised in the total extract of the three life cycle forms of the parasite. Antibody cross-reactivity was observed at approximately 138, 108, 86, 62, 45, 40 and 37 kDa in the three life cycle forms, and one reactive band around 65 kDa was exclusively found in amastigotes (Fig. 1A). It is important to highlight that *T. cruzi* calpains have *in silico* molecular masses ranging from 60 to 150 kDa, and the lower molecular mass proteins found here may be due to the autolytic conversion that calpains suffer in the presence of calcium.^1^ Moreover, the 65-kDa reactive band identified in amastigotes may be the product of the sequence *TcCLB.504107.10*, which is 56.9 times more expressed in amastigotes in relation to epimastigotes, since they share the same predicted molecular mass and life stage-specific expression pattern. In *L. braziliensis*, the anti-tritryp-calpain antibody detected polypeptides migrating at approximately 70, 45 and 40 kDa that were strongly recognised, along with faint bands either in high (150 and 225 kDa) or in low (47 and 31 kDa) molecular masses.^14^ A slight reactivity with molecules at approximately 127, 63, 43 and 35 kDa were observed in Vero cells extracts incubated with anti-tritryp-calpain antibody (Fig. 1A), which could be justified by the conservation of the peptide sequence in calpain 3 and 12 from the African green monkey (*Chlorocebus aethiops*) genome from which this cell line is derived from.

In addition to Western Blotting analysis, we performed flow cytometric assays with purified cells from the three distinct life cycle forms. Our results indicate a higher expression of reactive calpains in amastigotes and trypomastigotes in comparison to epimastigotes in 0.05% Triton X-100-permeabilised parasites, which allows a higher intracellular labeling of calpains by the anti-calpain antibody (Fig. 1B). By means of the calculation of the variation index of the mean of fluorescence intensity (MFI), it was possible to evaluate a global shift in antibody-recognised calpains from each life cycle form. Amastigote forms present a significantly higher amount of anti-tritryp-calpain antibody-reactive molecules, followed by trypomastigotes, and a much less abundant labeling in epimastigotes (Fig. 1C). Until nowadays, there is no report regarding the global calpain abundance among *T. cruzi* life cycle forms.

**Fig. 2:**
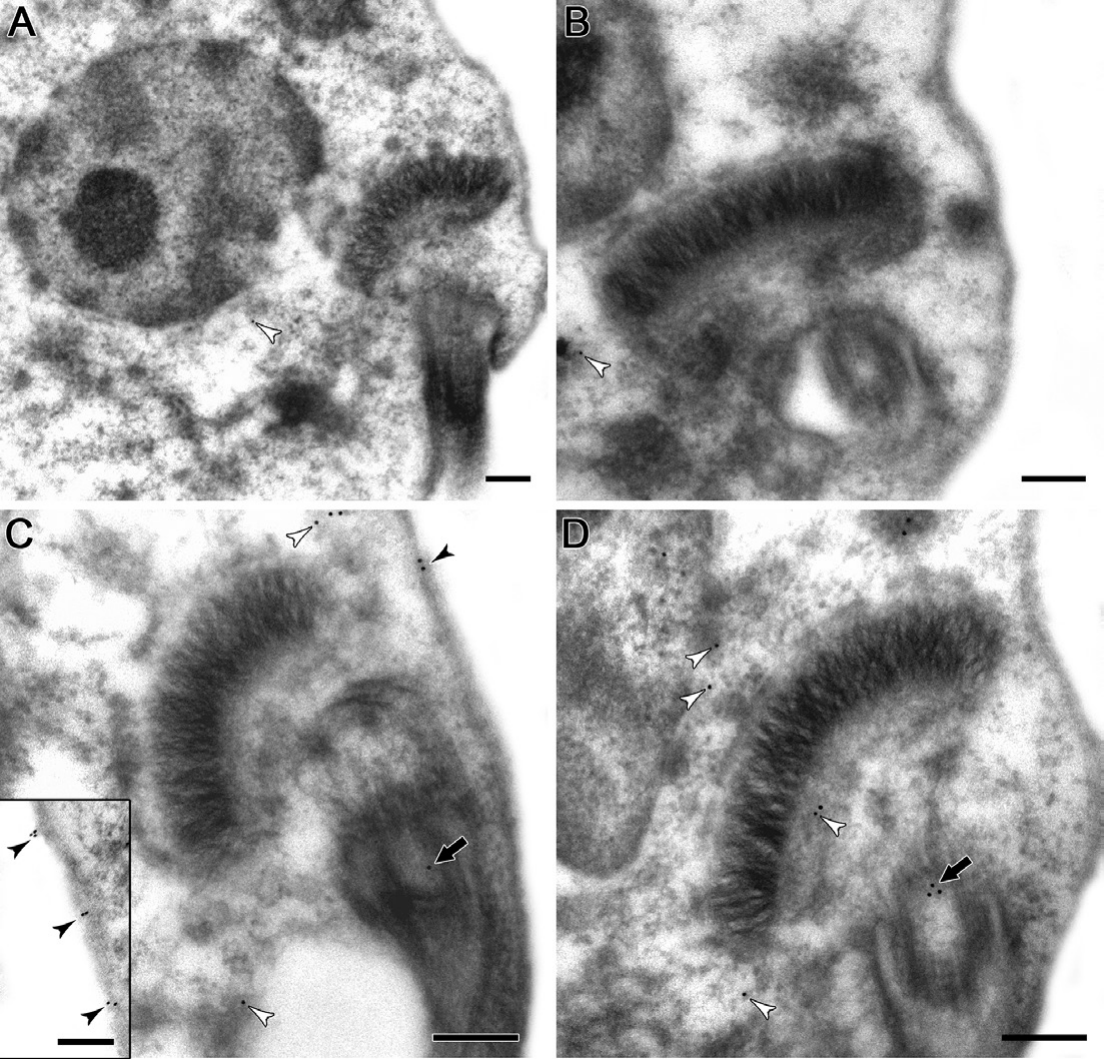
ultrastructural immunocytochemistry of calpains in *Trypanosoma cruzi* epimastigotes. The labelling was performed in ultrathin sections incubated with anti-trytrip-calpain antibody for 2 h, and subsequent incubation with secondary antibody conjugated to gold particles (10 nm) for 1 h. (A, B) The omission of the primary antibody pointed to the presence of rare unspecific labelling in the cytoplasm (white arrowheads). (C, D) Epimastigotes incubated with the anti-calpain antibody showed the labelling in plasma membrane (black arrowheads), in the whole cytoplasm (white arrowheads) and in the flagellum (black thick arrows). In detail, the labelling in the parasite surface (C, Inset). Bars: 0.2 μm. Inset Bar: 0.1 μm.

**Fig. 3:**
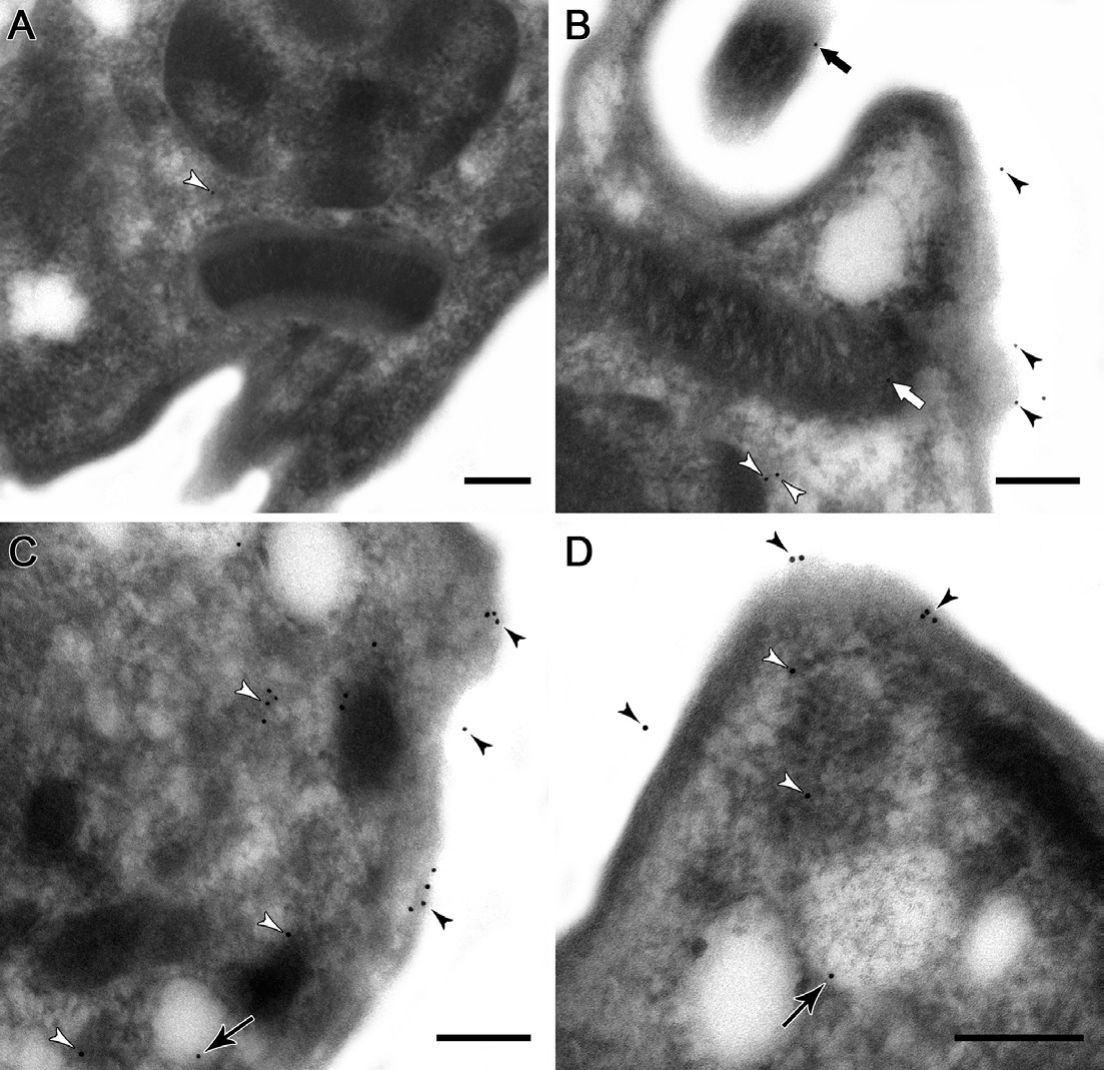
ultrastructural immunocytochemistry of calpains in *Trypanosoma cruzi* amastigotes. The labelling was performed in ultrathin sections incubated with anti-trytrip-calpain antibody for 2 h, and subsequent incubation with secondary antibody conjugated to gold particles (10 nm) for 1 h. (A) The omission of the primary antibody pointed to the presence of rare unspecific labelling in the cytoplasm (white arrowheads). (B, C, D) Amastigotes incubated with the anti-calpain antibody showed the labelling in plasma membrane (black arrowheads), in the whole cytoplasm (white arrowheads), in the paraflagellar rod (black thick arrows), in vesicles membranes (black arrows) and in rare unspecific labelling in the kinetoplast (white thick heads). Bars: 0.2 μm.

**Fig. 4:**
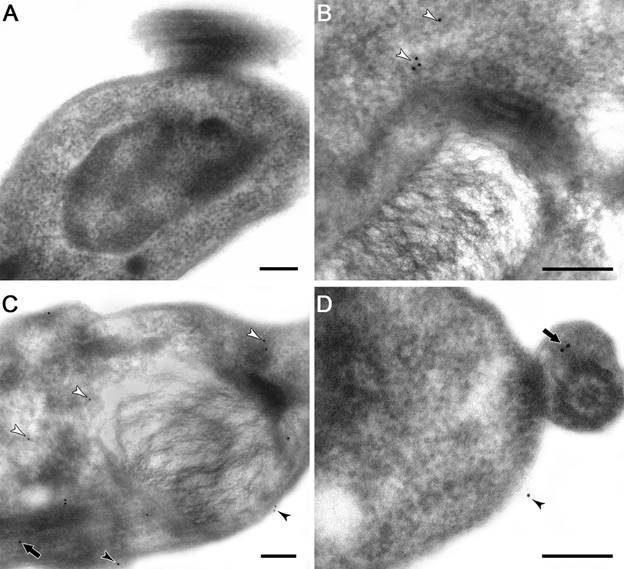
ultrastructural immunocytochemistry of calpains in *Trypanosoma cruzi* trypomastigotes. The labelling was performed in ultrathin sections incubated with anti-trytrip-calpain antibody for 2 h, and subsequent incubation with secondary antibody conjugated to gold particles (10 nm) for 1 h. (A, B) No unspecific labelling was detected in the absence of the primary antibody. (B, C, D) Trypomastigotes incubated with the anti-calpain antibody showed the labelling in plasma membrane (black arrowheads), in the whole cytoplasm (white arrowheads) and in the paraflagellar rod (black thick arrows). Bars: 0.2 μm.

*Ultrastructural localisation of calpains in* T. cruzi *epimastigotes, amastigotes and trypomastigotes* - It is well known that mammalian calpains are involved on calcium-regulated cellular functions such as apoptosis, differentiation, proliferation and cellular signaling, which are consistent with a cytoplasmic localisation.^1^ In *T. brucei* procyclic and bloodstream trypomastigotes, calpains have been shown to be mainly cytoplasmic,^21^ ^,^ ^22^ ^,^ ^26^ as well as in *T. cruzi* epimastigotes.^8^ Here, we performed ultrastructural immunolabeling with the anti-tritryp-calpain antibody to determine the cellular localisation of *T. cruzi* calpains in the three parasite forms. Our results pointed to the distribution of calpains throughout the cytoplasm, plasma membrane, and parasite flagellum in epimastigotes (Fig. 2B-D). Similar to flow cytometry analysis, a higher level of reactive molecules was detected in amastigotes. Calpains from this form were identified at the plasma membrane, cytoplasm, flagellum, membrane vesicle and rare nonspecific kinetoplast labeling (Fig. 3B-D). Trypomastigotes presented, in addition to the plasma membrane and cytoplasm labeling (Fig. 4B-C), a flagellar antibody-labeling, which strongly suggests that the FAZ is the detected region of the flagella (Fig. 4D). The repeat-rich H49/calpains described by Galetović and co-workers^10^ are also located at paraflagellar rod in trypomastigotes. Untreated anti-calpain controls reported a rare nonspecific labeling inherent of the technique (Figs 2A, 3A, 4A), as previously reported.^14^

In 2010, Liu and co-workers^22^ obtained similar results in an immunofluorescence analysis employing specific anti-calpain antibodies in *T. brucei*, reporting calpain-labeling in cytoplasm, flagellum and on the cell body periphery. In order to better understand the flagellar end calpain location, the authors performed site-specific mutations and verified the loss of cellular protein localisation when at least 35 amino acid residues were deleted from the C-terminal. Although no *in silico* data reported sequence motifs at the C-terminal end indicating this flagellar localisation, the peptide sequence of this protein has an accumulation of basic residues and isoelectric point of 9.7, similar as its ortholog in *T. cruzi*, the calpain *TcCLB.506563.200* studied here (pI = 10.6). The authors also reported that no ortholog of this calpain is present in the annotated *Leishmania* genomes, and that the location of this protein at the tip of the flagellum suggests a possible involvement in parasite sensory functions.^22^ Moreover, a repeat-rich calpain of *T. brucei*, called CalpGM6, has been identified as essential for elongation process and flagella cellular localisation during the multiplication of procyclic forms in axenic culture.^27^ CalpGM6 RNA*i* silencing reduced parasite proliferation rate and induced epimastigote-like forms formation, with central flagellum located near the nucleus and almost completely free. In addition, the ultrastructural analysis performed in the study allowed to observe shortening and structural disorganisation of the FAZ, which led the authors to suggest the association of this calpain with the parasite structural organisation.^27^

*In conclusion* - The *T. cruzi* calpains have a wide range of domain arrangements, present differential expression patterns among *T. cruzi* life cycle forms, with a global shift towards amastigotes, and are distributed in the cytoplasm, membranes and flagellum in all life cycle forms of the parasite. It has been previously shown the chemotherapeutic potential of the calpain inhibitor MDL28170 against the different life cycle forms of *T. cruzi*,^8^ and against a broad range of *Leishmania* species.^14^ ^,^ ^28^ The diverse and extensive profile of calpains expressed by *T. cruzi* suggest that these molecules play crucial role in the parasite life cycle. Curiously, calpain activity has never been biochemically detected so far, although sequences with the conserved catalytic triad are transcribed to RNA. Further studies will help to increase the knowledge about this fascinating peptidases family and answer the puzzle: Are we unable to measure calpain activity or biological calpain functions are activity-independent in these parasites?
