# Supplementary material for: Expression and cellular localisation of Trypanosoma cruzi calpains
Source: Mem Inst Oswaldo Cruz. 2020 Oct 12;115:e200142. doi: 10.1590/0074-02760200142 (PMC7552305; doi:10.1590/0074-02760200142)
Supplement: Supplementary file 1 [file 1678-8060-mioc-115-e200142-s.pdf]

TABLE I

Gene-specific primers designed for quantitative polymerase chain reaction (qPCR) gene expression analysis

Sequence ID	Forward primer	Reverse primer	e-value
TcCLB.511307.10	TGGATGATGCTGGGCGGGAG	CCTCCTTGCAGCGCTCCTCT	$9e^{-23}$
TcCLB.511727.30	ATGGAGCCCGTTCATCCCCC	CCGCTGGGTGCTTTCGCTTG	$7e^{-21}$
TcCLB.509237.140	AGGGCGGTGGTGTCTGCTAC	TCCGCCTTCTGCTCAACCCG	$1e^{-20}$
TcCLB.509237.120	GCTTAGGGCTGGAGCTGGGT	GCCGCATTCCCCACACACAC	$2e^{-20}$
TcCLB.508999.200	CGTAGTCCGGTGGAGGTGGG	TCCGTTTGGCACGACACCGT	$2e^{-41}$
TcCLB.510337.20	AGGTTTGCCCACAGCAGCGA	AATCGAGAGGCGGGACACCC	$1e^{-24}$
TcCLB.504107.10	TCCGCCACTTGGACCCATCC	CACCAGCAGGAGTTCGGCAC	$1e^{-28}$
TcCLB.511269.70	TCCGTGCTGGGTGAATATCGGC	CTCGGGAGATGCTGCAAACGC	$4e^{-24}$
TcCLB.508999.190	TACCCAGACGTGGCTGAGGC	AAAGGAAACGCCCCCACCGT	$2e^{-30}$
TcCLB.511329.10	TGCAGAGTCAAGGGACCGGG	AGTACAGGGCACCACCAGCC	$7e^{-39}$
TcCLB.508999.230	CATTCCCCGCACCACATCGG	ACTCCTCGTTGCAGGAGCCC	$3e^{-23}$
TcCLB.511507.70	GGAAGAAACAGTGCGGGCGG	AGCGTGTGTCAGGAACGGCA	$2e^{-30}$
TcCLB.511333.4	CGACACGACGAGTACCCGCA	AACGGCGACGTCATCCGCCA	$5e^{-21}$
TcCLB.511847.10	GTCGAGAACGCCTTGCTGCT	TGCCGAGGGATTTGTCCTGC	$1e^{-20}$
TcCLB.506563.200	TTGGGGCACCGACCTTTGTC	ACGCCCACACATCATCCTTAGC	$7e^{-24}$
TcCLB.509013.19	AACAGCGAGTGGGGTGAGGG	GCTTGTGTCGTTGGTCCGGC	$2e^{-40}$
TcCLB.506943.50 (GAPDH)	GTGCGGCTGCTGTCAACAT	AAAGACATGCCCGTCAGCTT	$6e^{-22}$
AF232214 (18S, Mathieu-Daudé, 2007)	GTGTGACCAAAGCAGTCATTCG	TGTCACACTGCCCCATAATCTC	$3e^{-33}$

Glyceraldehyde-3-phosphate dehydrogenase (GAPDH) and 18S were used as endogenous controls. Each primer was first evaluated for the production of a single product from cDNA template through conventional polymerase chain reaction (PCR) followed by gel agarose analysis. The amplified products were purified and sequenced in a Sanger sequencer as described in Materials and Methods. The sequences were aligned against *Trypanosoma cruzi* genome to assure that each primer was targeting the sequence of interest, as demonstrated by the e-value score.

CC BY

TABLE II

Calpain sequences retrieved from *Trypanosoma cruzi* genome

ID TritrypDB	Esmeraldo	Chromosome	Predicted molecular mass	Domain architecture	Conserved catalytic triad	Consensus peptide sequence (LEKAYAKLHGSY)
TcCLB.509003.30	no	9	14.87	DUF1935	----------------	----------------
TcCLB.506563.110	yes	9	16.74	DUF1935	----------------	----------------
TcCLB.509001.40	no	9	16.68	DUF1935	----------------	----------------
TcCLB.508999.260	no	9	14.65	DUF1935	----------------	----------------
TcCLB.510957.9	yes	9	40.31	DUF1935	----------------	----------------
TcCLB.506563.130	yes	9	18.88	DUF1935	----------------	----------------
TcCLB.509003.40	no	9	15.4	DUF1935	----------------	----------------
TcCLB.506563.50	yes	9	15.18	DUF1935	----------------	----------------
TcCLB.509003.60	no	9	15.16	DUF1935	----------------	----------------
TcCLB.506563.120	yes	9	18.7	DUF1935	----------------	----------------
TcCLB.506563.170	yes	9	14.67	DUF1935	----------------	----------------
TcCLB.506563.70	yes	9	15.4	DUF1935	----------------	----------------
TcCLB.506563.90	yes	9	22.65	DUF1935	----------------	----------------
TcCLB.509003.20	no	9	22.72	DUF1935	----------------	----------------
TcCLB.509001.30	no	9	18.85	DUF1935	----------------	----------------
TcCLB.509001.20	no	9	34.38	DUF1935	----------------	----------------
TcCLB.506563.79	yes	9	14.9	DUF1935	----------------	----------------
TcCLB.506563.180	yes	9	15.86	DUF1935	----------------	----------------
TcCLB.508999.250	no	9	15.83	DUF1935	----------------	----------------
TcCLB.508675.29	yes	11	12.81	DUF1935	----------------	----------------
TcCLB.506983.39	no	11	12.85	DUF1935	----------------	----------------
TcCLB.506983.48	no	11	12.85	DUF1935	----------------	----------------
TcCLB.511335.9	yes	31	20.94	DUF1935	----------------	----------------
TcCLB.509237.151	no	31	29.49	DUF1935	----------------	----------------
TcCLB.510091.119	yes	40	15.6	DUF1935	----------------	----------------
TcCLB.511333.10	yes	31	24.7	fragmented CysPc	----------------	low similarity
TcCLB.511441.20	yes	39	19.13	CysPc	----------------	LEKAYAKFYTGY
TcCLB.503855.40	yes	8	95.23	DUF1935, CysPc	yes	LQKAFAKLNGSY
TcCLB.510337.20	no	8	95.4	DUF1935, CysPc	yes	LQKAFAKLNGSY
TcCLB.506563.190	yes	9	94.62	DUF1935, CysPc	----------------	VEKAYAKLHGNY
TcCLB.508999.200	no	9	78.11	DUF1935, CysPc	----------------	LQKAYAKIHGGY
TcCLB.506563.210	yes	9	78.3	DUF1935, CysPc	----------------	LEKAYAKLHGSY
TcCLB.506563.200	yes	9	82.64	DUF1935, CysPc	----------------	LEKAYAKLHGSY
TcCLB.508999.220	no	9	82.47	DUF1935, CysPc	----------------	LEKAYAKLHGSY
TcCLB.508999.190	no	9	95.78	DUF1935, CysPc	----------------	LEKAFAKLHGSY
TcCLB.508999.230	no	9	92.63	DUF1935, CysPc	----------------	VEKAYAKLHGNY
TcCLB.506227.130	yes	14	120.56	CysPc, CBSW	yes	MEKAFVKLCGGSY
TcCLB.511847.10	no	14	98.6	CysPc, CBSW	yes	MEKAFVKLCGGSY
TcCLB.511307.10	yes	17	150.32	KISC, 3×ARM, CBSW	----------------	low similarity
TcCLB.507087.40	no	17	150.32	KISC, 3xARM, CBSW	----------------	low similarity
TcCLB.508555.70	yes	31	137.88	DUF1935, CysPc	----------------	LEKAYAKALGSY
TcCLB.509237.140	no	31	84.37	DUF1935, CysPc	----------------	LEKAYAKALGSY
TcCLB.508555.50	yes	31	80.96	DUF1935, CysPc	----------------	VEKAYAKVHGSY
TcCLB.509237.120	no	31	81.06	DUF1935, CysPc	----------------	VEKAYAKVHGSY
TcCLB.511333.4	yes	31	80.79	DUF1935, CysPc	----------------	LQKAYAKLHGSY
TcCLB.508555.60	yes	31	89.82	DUF1935, CysPc	----------------	LQKAYAKLHGSY
TcCLB.509237.130	no	31	88.68	DUF1935, CysPc	----------------	LQKAYAKLHGSY
TcCLB.511329.10	yes	31	64.88	CysPc	----------------	LEKAYAKVHHSY
TcCLB.511507.70	no	32	115.98	RNI-like, CysPc, CBSW	----------------	LEKALAKLNGGY
TcCLB.503909.84	yes	32	116.16	RNI-like, CysPc, CBSW	----------------	LEKALAKLNGGY
TcCLB.511727.30	no	35	161.78	CysPc	----------------	low similarity
TcCLB.509013.19	yes	39	135.52	CysPc, CBSW	----------------	LEKAYAKFYTGY
TcCLB.504107.10	yes	39	65.54	CysPc, CBSW	----------------	LEKAYAKFYTGY

ID TritrypDB	Esmeraldo	Chromosome	Predicted molecular mass	Domain architecture	Conserved catalytic triad	Consensus peptide sequence (LEKAYAKLHGSY)
TcCLB.507829.10	yes	40	85.46	CysPc	----------------	VEKAFAKLYGGY
TcCLB.511269.70	no	40	88.15	RNI-like, CysPc, CBSW	----------------	VEKAFAKLYGGY
TcCLB.506493.90	yes	5	179.7	RNI-like, CBSW, CysPc, RPT1	----------------	LEKAYAKHHRCY
TcCLB.510121.170	no	5	179.71	RNI-like, CBSW, CysPc, RPT1	----------------	LEKAYAKHHRCY
TcCLB.509013.10	yes	39	154.68	RPT1, CysPc, CBSW	----------------	LEKAYAKFVGGY
TcCLB.505985.9	no	39	200.83	CysPc, RPT3, RPT2	----------------	low similarity
TcCLB.511441.10	yes	39	145.25	2×RPT1, CysPc	----------------	LEKAYAKFVGGY
TcCLB.506925.550	yes	39	167.65	CysPc, CBSW, RPT1	----------------	LEKAYAKFYTGY
TcCLB.511445.10	yes	39	132.88	2×RPT1, CysPc, CBSW	----------------	LEKAYAKFVGGY
TcCLB.506721.30	no	39	519.41	CysPc, CBSW, CysPc, RPT2, CysPc, CBSW	-	LEKAYAKFVGGY

The ID of the calpain sequences was retrieved from *T. cruzi* CL Brener strain genome (Genebank ID 25). Based on the domain architecture, the sequences were ordered by their chromosome location. The predicted molecular mass was calculated in http://www.bioinformatics.org/sms/prot_mw.html. The identity and similarity of the conserved immunogenic consensus sequence in the CysPc domain are shown: bold letters indicate conserved amino acid, residues with strong similar properties are in italics and underlined and residues with weak similar properties are in grey. Calpain sequences that contained the CysPc domain but an overall identity lower than 75% with the consensus peptide were designated as "low similarity", and "----------" indicates the absence of the sequence. Calpain sequences ID in bold highlight the selection for differential gene expression analyses. ARM: armadillo/beta-catenin-like repeats; CBSW: calpain-type alpha-beta-sandwich domain; CysPc: proteolytic core domain; KISC: kinesin domain; DUF1935: conserved N-terminal calpain-related domain from trypanosomatids; RPT: repeated domain found in de-ubiquitinating proteins; RNI-like: regular structure consisting of similar repeats; 2× and 3× indicate the number of times that a domain appears in the sequence. Fragmented CysPc stands for short amino acid sequences from the catalytic domain.
